# Profiling and Molecular Mechanism Analysis of Long Non-Coding RNAs and mRNAs in Pulmonary Arterial Hypertension Rat Models

**DOI:** 10.3389/fphar.2021.709816

**Published:** 2021-06-29

**Authors:** Shiqiang Hou, Dandan Chen, Jie Liu, Shasha Chen, Xiaochun Zhang, Yuan Zhang, Mingfei Li, Wenzhi Pan, Daxin Zhou, Lihua Guan, Junbo Ge

**Affiliations:** ^1^ Department of Cardiology, Zhongshan Hospital, Fudan University, Shanghai Institute of Cardiovascular Disease, Shanghai, China; ^2^ National Clinical Research Center for Interventional Medicine, Shanghai, China; ^3^ Department of Thoracic Surgery, Shanghai East Hospital, Tongji University School of Medicine, Shanghai, China

**Keywords:** pulmonary arterial hypertension, lung, long non-coding RNA, messenger RNA, inflammatory immune disease

## Abstract

Pulmonary arterial hypertension (PAH) is an immune-mediated disease with poor prognosis and associated with various inflammatory immune diseases. In fact, its pathogenesis is far from clear. Although long non-coding RNAs (lncRNAs) have been implicated in PAH, the molecular mechanisms remain largely unknown. For the first time, in lungs of monocrotaline-induced PAH rat models, we simultaneously detected the expression profiles of lncRNAs and mRNAs by high-throughput sequencing, and explored their roles with bioinformatics analysis and cell assay to discover more potential pathogenesis about PAH. Our data identified that a total of 559 lncRNAs and 691 mRNAs were differentially expressed in lungs during the pathogenesis of PAH. Gene Ontology (GO) and Kyoto Encyclopedia of Genes and Genomes (KEGG) analyses demonstrated that these dysregulated lncRNAs and mRNAs participated in important biological processes and pathways of PAH, among which inflammatory and immune responses represented the chief enriched pathway. The lncRNA-mRNA co-expression network was developed to uncover the hidden interactions between lncRNAs and mRNAs. Further, the expression levels of lncRNAs (NONRATT018084.2, NONRATT009275.2, NONRATT007865.2, and NONRATT026300.2) and mRNAs (LGALS3, PDGFC, SERPINA1, and NFIL3) were confirmed using quantitative real-time PCR. In the end, lncRNA NONRATT009275.2 could facilitate macrophage polarization to M2 type and be involved in inflammatory immune response. In conclusion, this study provided candidate drug targets and potential roles on lncRNAs in the pathogenesis of PAH, and several key regulatory genes were identified, which laid the initial foundation for further mechanism study in PAH.

## Introduction

Pulmonary arterial hypertension (PAH) is an immune-mediated progressive disease characterized by increases in pulmonary vascular resistance and pulmonary artery pressure that ultimately lead to right ventricular failure and death (Humbert et al., 2004). It is a common complication of many inflammatory immune diseases, such as systemic lupus erythematosus and rheumatoid arthritis (Qu et al., 2021; Vonk et al., 2021). In past 2 decades, the long-term survival of PAH patients has improved to a certain extent (Thenappan et al., 2010). Current drug treatments are mainly based on three critical signaling pathways containing endothelin, prostacyclin, and nitric oxide. However, they have not resulted in an effective strategy which could reverse pulmonary vascular remodeling (PVR) and prevent deterioration and the need for a lung transplant (Galiè et al., 2016). As a severe and debilitating cardiopulmonary disease, PAH still poses a huge clinical and economic burden, and the hospital mortality does not decrease (Anand et al., 2016). Therefore, it is urgent to identify new potential therapeutic targets for PAH.

An expanding body of knowledge has related the structural abnormalities of pulmonary vascular wall to PAH pathogenesis, including pathological growth of intimal, medial, and adventitial layers (Humbert et al., 2014). At present, many autoimmune diseases are known to cause PAH. Recent studies have focused on inflammatory cells and their mediators as pivotal contributors to PVR and dysfunction in PAH. Perivascular inflammatory infiltrates occur in patients with all forms of PAH as well as in animal models, comprising T- and B-lymphocytes, macrophages, dendritic cells, and mast cells (Berghausen et al., 2019). Moreover, serum levels of multiple inflammatory cytokines and chemokines are also elevated (Anwar et al., 2016). The above evidences suggest that PAH is an inflammatory immune disease, but the related molecular mechanisms are far from clear. Besides PVR and immune microenvironment, additional research directions also need to be further explored. Hence, we chose lung tissues for sequencing to have a holistic view of gene expression about PAH, and find more possible pathogenesis.

Long non-coding RNAs (lncRNAs) are a group of non-coding RNAs (ncRNAs) that have transcripts of greater than 200 nucleotides in length (Mercer et al., 2009). It has been reported that lncRNAs are involved in almost all biological procedures, and these effects occur through epigenetic regulation, translational regulation, transcriptional regulation, and post-transcriptional regulation (Guttman and Rinn, 2012; Kopp and Mendell, 2018). Some studies reveal that lncRNAs participate in cardiovascular diseases, as a result, which have been proposed to be new targets for pharmaceutical intervention (Poller et al., 2018; Turkieh et al., 2019; Zahid et al., 2020; Wu et al., 2021). Just in recent years, the mechanisms of lncRNAs in PAH are beginning to be vigorously investigated. Several lncRNAs could be linked to pulmonary artery smooth muscle cells (PASMCs) disorders and endothelial dysfunction by regulating proliferation and migration (Neumann et al., 2018; Su et al., 2018; Zhu et al., 2018; Gong et al., 2019). However, the present understandings on differential expression, pathophysiological function of lncRNAs, and potential interaction between lncRNAs and mRNAs in PAH, remain largely unknown.

In this study, we firstly performed high-throughput sequencing to simultaneously detect lncRNAs and mRNAs expression patterns of PAH in lungs from monocrotaline (MCT)-induced PAH rat models. Further, gene-ontology (GO) and pathway enrichment analyses were done to explore the roles of aberrantly expressed genes in the pathogenesis of PAH. We also constructed a co-expression network for the dysregulated lncRNAs and mRNAs, to expound the interactions between them. Finally, several critical lncRNAs and mRNAs, predicted by bioinformatics analysis, were verified by quantitative real-time PCR (qRT-PCR). And we used cell assay to preliminarily verify that lncRNA NONRATT009275.2 could facilitate macrophage polarization to M2 type and be involved in inflammatory immune response.

## Methods and Materials

### Animal Model

Four weeks old male Wistar rats (180–200 g) were purchased from the Shanghai Laboratory Animal Center (Chinese Academy of Sciences, Shanghai, China). All animal experiments were performed in accordance to the National Institutes of Health guide for the care and use of Laboratory animals, with the approval of the Institutional Animal Care and Use Committee of Fudan University (Shanghai, China). Rats were randomly divided into Control and monocrotaline-induced PAH (MCT-PAH) groups (*n* = 3). The PAH model was induced through subcutaneous injection in a single dose of MCT (60 mg/kg, Sigma-Aldrich, Merck Millipore, Germany). The control rats were injected with the same volume of normal saline. After 8 weeks post-MCT injection, rats were anesthetized and subjected to hemodynamic measurement to confirm PAH modeling success. The right ventricular systolic pressure and pulmonary artery pressure were recorded with a PowerLab data acquisition system using an ML110 pressure transducer (ADInstruments, New South Wales, Australia). After hemodynamic measurements, lung tissues were collected for further examination.

### RNA Isolation

Total RNAs were isolated from lung tissues with a mirVana miRNA Isolation Kit (Ambion, MA, United States), according to the manufacturer’s instructions. RNA integrity was evaluated by the Agilent 2100 Bioanalyzer (Agilent Technologies, Santa Clara, CA, United States). RNA quantity and quality were assessed using the NanoDrop 2000 (Thermo, Waltham, MA, United States).

### Library Construction and Sequencing

The library was constructed using the TruSeq Stranded Total RNA Library Prep Kit with Ribo-Zero Gold High Throughput (Illumina, San Diego, CA, United States) following the manufacturer’s protocols. Then, this library was sequenced on the Illumina sequencing platform (HiSeqTM 2500). All high-throughput sequencing programs were performed by OE Biotech (Shanghai, China). Aberrantly expressed lncRNAs and mRNAs between Control group and MCT-PAH group were identified with fold change (FC), *p* value and the false discovery rate (FDR) filtering.

### Gene Function Analysis

Gene ontology (GO) enrichment analysis of aberrantly expressed mRNAs was implemented to investigate functions according to the three following aspects: biological process, cellular component and molecular function. Kyoto Encyclopedia of Genes and Genomes (KEGG) analysis was performed to classify the biological pathway clusters covering the dysregulated mRNAs. A *p*-value <0.05 was considered statistical significance.

### Construction of the Long Non-Coding RNA-mRNA Co-Expression Network

In order to establish the co-expression network between aberrantly expressed lncRNAs and mRNAs, a Pearson correlation coefficient (PCC) statistic method was used to evaluate every aberrantly expressed lncRNA-mRNA combination. A PCC value >0.99 was considered statistically significant, and was retained for network construction using Cytoscape (version 3.4.0).

### Verification of Dysregulated RNA Expression by qRT-PCR

Total RNAs from lung tissues were isolated with a mirVana miRNA Isolation Kit (Ambion, MA, United States) in another three MCT-PAH rats and control rats. The cDNA used for lncRNA and mRNA analysis was synthesized by using a PrimeScript RT reagent Kit (Takara-Bio, Shanghai, China). The qRT-PCR analysis was carried out using All-inOne qPCR Mix (GeneCopoeia, Rockville, MD, United States) and performed with ABI 7500 Fast Real-Time PCR System (Applied Biosystems) following the manufacturer’s instructions. Glyceraldehyde 3-phosphate dehydrogenase (GAPDH) was employed as an internal control. The primers were listed in Supplementary Table S1 **.**

### Bone Marrow-Derived Macrophages Isolation and Culture

BMDMs were isolated as previously described (Liu et al., 2015). Femurs were obtained from 4-week-old male Wistar rats. BMDMs were co-cultured with 10% FBS, 30% L929, and 60% DMEM in suspension. Then IL-4 (20 ng/ml; PEPROTECH, United States) was used to treat the cells. qRT-PCR was performed with production of 4 h treatment and flow cytometry was performed using production of 16 h treatment.

### Cell Transfection and mRNA Detection

LncRNA NONRATT009275.2 was constructed into pIRES2-EGFP plasmid (GENE, Shanghai, China). The vector was used as control. These plasmids were transfected into BMDMs using lipofectamine 3000 (Life Technologies) according to the manufacturer’s instructions. To be specific, BMDMs were seeded into six-well plates. When cell confluency reached approximately 70%, cells were then incubated in DMEM containing 0.5% FBS. After serum starvation for 6 h, cells were washed with PBS for two times and added 1 ml of Opti-MEM medium. Meanwhile, 8 μl of Lipofectamine 3000 were diluted in tube 1 containing 125 μl Opti-MEM medium. And 3 μg of plasmid and 6 μl of P3000 enhancer were diluted in tube 2 containing 125 μl Opti-MEM medium. Then, tube 2 solution was added to tube 1 and mixed well. After incubation at room temperature for 15 min, the mixture was added to cells. After incubation for 6 h, cells were added with fresh complete medium for further incubation for 18 h. Afterward, the transfected cells were treated IL-4 (20 ng/ml; PEPROTECH, United States). qRT-PCR was performed with production of 4 h treatment. The primers were listed in Supplementary Table S1.

### Cell Staining and Flow Cytometry

BMDMs were stained with fluorescence conjugated antibodies against F4/80 (BioLegend, United States), CD206 (BioLegend, United States). Data were acquired with a FACScan flow cytometer (BD Biosciences, United States). Gates were set on the population of mono-nuclear macrophages. And FlowJo Software (TreeStar, United States) was used for analysis.

### Statistical Analysis

Data were represented as the mean ± standard deviation (mean ± SD). Statistical significance was evaluated with an unpaired two-tailed Student’s *t*-test for 2 independent groups. Statistical analyses were performed with GraphPad prism 6.0 (GraphPad, San Diego, CA, United States). A *p*-value <0.05 was considered statistical significance.

## Results

### Aberrant Expression Profiles of Long Non-Coding RNAs and mRNAs

**FIGURE 1:**
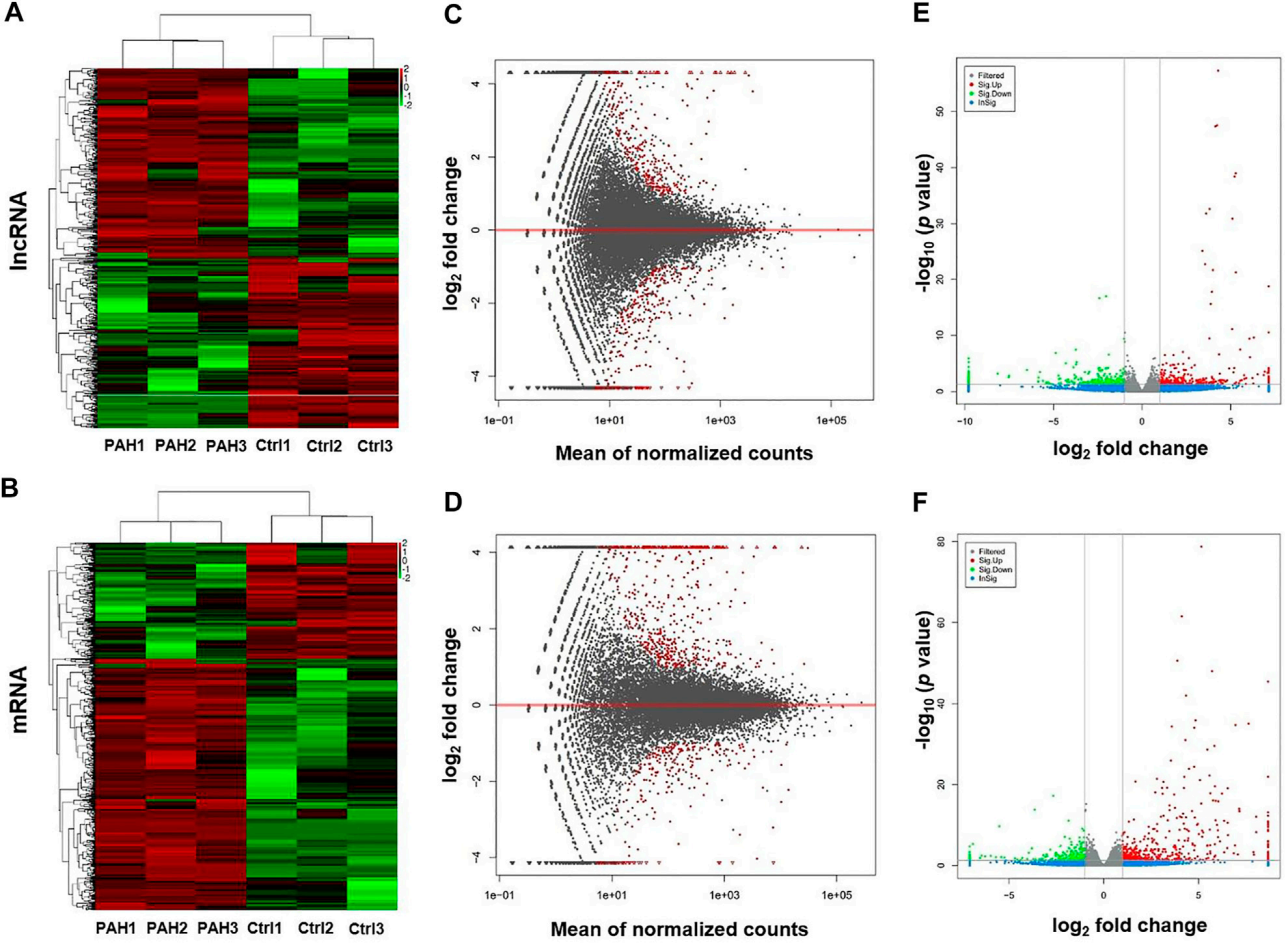
Profiling of lncRNAs and mRNAs between PAH group and control group. **(A,B)** Heat maps showed the hierarchical clustering of differential expression in lncRNAs and mRNAs between PAH group (PAH) and control group (Ctrl). **(C,D)** MA plots showed the differential expression of lncRNAs and mRNAs between two groups. Differential expression was indicated as “red”. **(E,F)** Volcano plots were depicted to visualize the up-regulated and down-regulated lncRNAs and mRNAs between two groups. Up-regulated expression was indicated as “red”, and down-regulated expression was indicated as “green”.

High-throughput sequencing was employed to assess the expression profiles of lncRNAs and mRNAs in MCT-PAH rats and control rats, as a result, which detected 31,275 lncRNAs and 28,635 mRNAs. A total of 559 differentially expressed lncRNAs (FC > 2.0, *p* < 0.05), including 295 up-regulated and 264 down-regulated lncRNAs, were identified between MCT-PAH rats and control rats. Moreover, 473 up-regulated mRNAs and 218 down-regulated mRNAs were determined between two groups (FC > 2.0, *p* < 0.05). Visualization using hierarchical clustering of the differential expression of lncRNAs (Figure 1A) and mRNAs (Figure 1B) displayed significant variations in heat maps. The top 10 up- and down-regulated lncRNAs and mRNAs were displayed in Tables 1, 2, respectively. NONRATT033431.1 (FC = 141.460) and NONRATT002354.2 (FC = 0.001) were the most significantly up- and down-regulated lncRNAs. RT1-S2 (FC = 116.382) and Ttc23 (FC = 0.007) were the most significantly up- and down-regulated mRNAs in MCT-PAH rats compared with controls. MA plots and volcano plots were constructed to display the distribution for aberrantly expressed lncRNAs (Figures 1C,E) and mRNAs (Figures 1D,F) among the samples, respectively. The characteristics of dysregulated lncRNAs were shown in Supplementary Figure S1.

**TABLE 1:** Top 10 significantly differential expressed lncRNAs identified by high-throughput sequencing.

Up-regulated			Down-regulated		
lncRNAs	*p* value	Fold change	lncRNAs	*p* value	Fold change
NONRATT033431.1	0.000	141.460	NONRATT002354.2	0.002	0.001
NONRATT011934.2	0.000	134.949	NONRATT013281.2	0.001	0.004
NONRATT017032.2	0.000	121.137	NONRATT006789.2	0.003	0.005
NONRATT023671.2	0.001	116.557	NONRATT016013.2	0.002	0.006
NONRATT019381.2	0.001	92.126	NONRATT030453.2	0.000	0.011
NONRATT033423.1	0.000	78.938	NONRATT024649.2	0.015	0.017
NONRATT016587.2	0.039	77.800	NONRATT011637.2	0.020	0.017
NONRATT015297.2	0.004	74.805	NONRATT012347.2	0.003	0.017
NONRATT033430.1	0.000	68.169	NONRATT022660.2	0.000	0.018
NONRATT025409.2	0.000	66.438	NONRATT004729.2	0.013	0.021

**TABLE 2:** Top 10 significantly differential expressed mRNAs identified by high-throughput sequencing.

Up-regulated			Down-regulated		
mRNAs	*p* value	Fold change	mRNAs	*p* value	Fold change
RT1-S2	0.001	116.382	Ttc23	0.004	0.007
Ighg	0.000	93.277	Atp1b1	0.000	0.008
RGD1563231	0.000	81.799	Fosb	0.013	0.011
AABR07065699.3	0.000	75.646	LOC100911581	0.005	0.013
Zbtb44	0.045	65.602	Synj1	0.004	0.016
Ighm	0.000	60.140	Emr4	0.009	0.019
LOC100360581	0.039	77.800	Fam8a1	0.000	0.022
Hspbap1	0.007	57.390	Atf3	0.008	0.024
Sult1c2a	0.036	54.683	Fos	0.010	0.026
RGD1565617	0.000	48.433	Sugt1	0.033	0.043

### Functional Prediction of Aberrantly Expressed mRNAs

**FIGURE 2:**
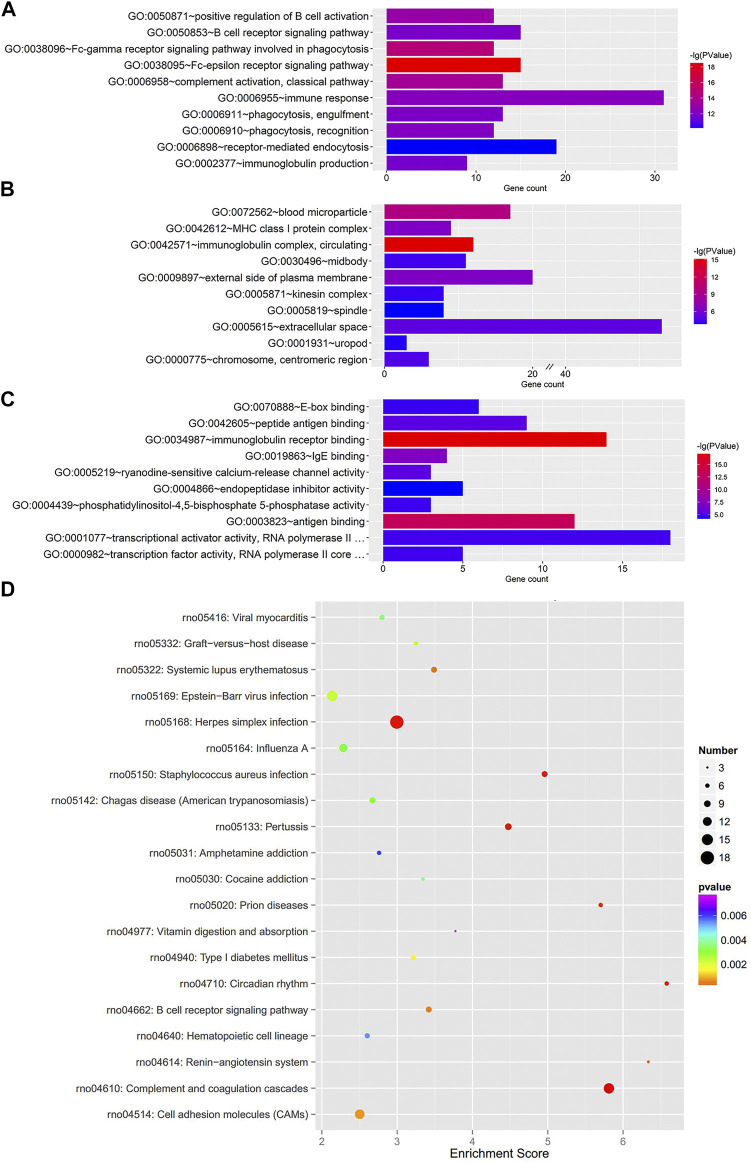
GO enrichment and KEGG pathway analyses for the total dysregulated mRNAs. Top 10 enriched GO terms of total dysregulated mRNAs were presented according to **(A)** biological process, **(B)** cellular component and **(C)** molecular function, respectively. **(D)** KEGG pathway analysis of the total dysregulated mRNAs in PAH.

To further clarify the functional roles of these dysregulated mRNAs in the MCT-PAH rats, we performed GO enrichment and KEGG pathway enrichment analyses. The GO analysis showed terms related to three parts: biological process (BP), cellular component (CC), and molecular function (MF). The total differentially expressed mRNAs were significantly enriched in Fc-epsilon receptor signaling pathway (BP), immunoglobulin complex-circulating (CC), and immunoglobulin receptor binding (MF) (Figures 2A–C). Furthermore, KEGG pathway analysis was conducted to predict the potential pathways. Various important pathways involved in PAH, including Complement and coagulation cascades (rno04610), Circadian rhythm (rno04710), Renin-angiotensin system (rno04614), Systemic lupus erythematosus (rno05322), and B cell receptor signaling pathway (rno04662), were significantly enriched in the dysregulated mRNAs (Figure 2D). In addition, the up- and down-regulated mRNAs were also separately subjected to GO and KEGG enrichment analyses, which were shown in Supplementary Figures S2, S3, respectively. Similar to the total differentially expressed mRNAs, the up-regulated mRNAs were also enriched in immune and inflammatory responses. Apart from this, for down-regulated mRNAs, cell proliferation was another enriched function.

### The Long Non-Coding RNA-mRNA Co-Expression Network

**FIGURE 3:**
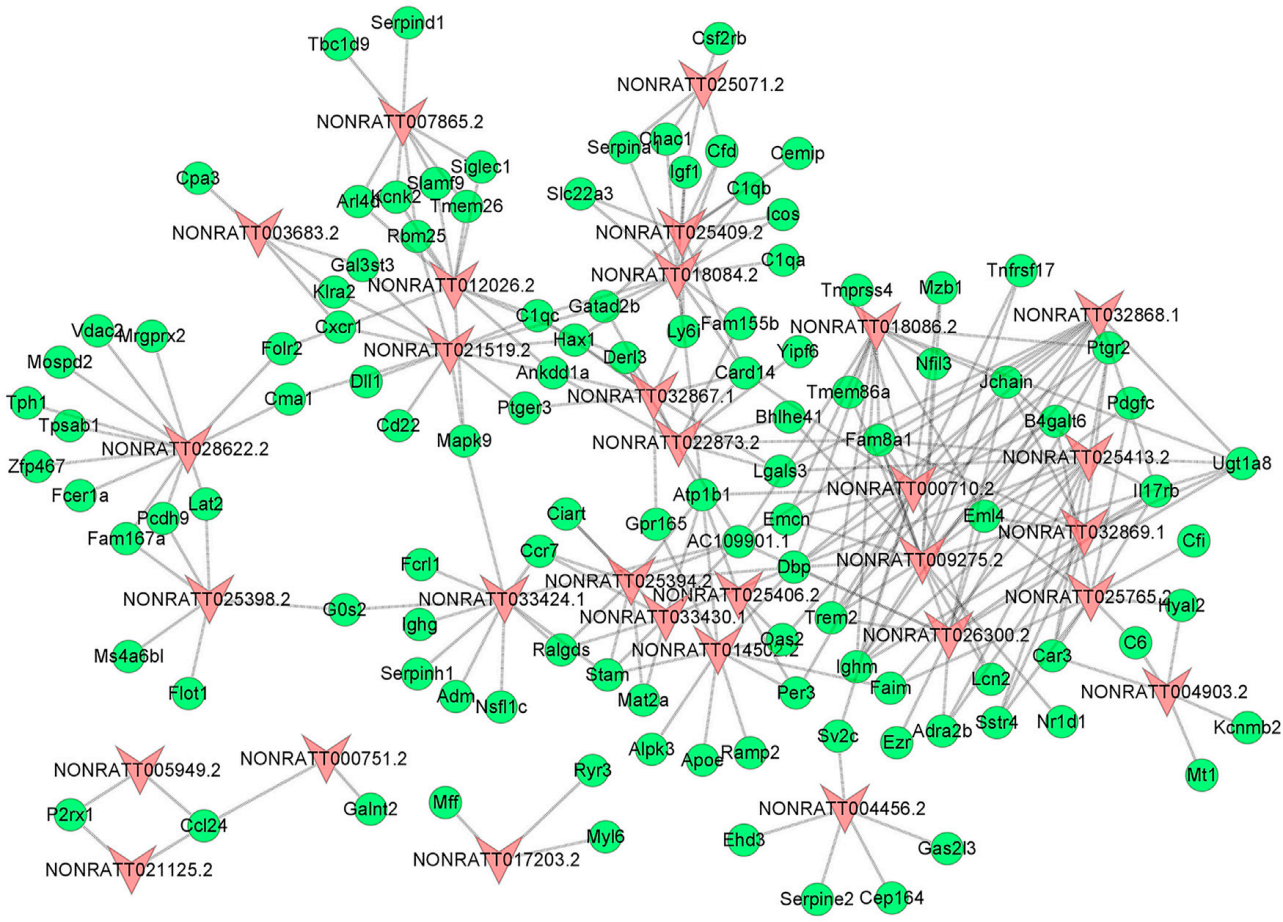
The lncRNA-mRNA Co-Expression Network. The red triangle nodes represented differentially expressed lncRNAs, and the green oval nodes represented differentially expressed mRNAs. The edges showed the interactions between lncRNAs and mRNAs.

To uncover the hidden interactions between lncRNAs and mRNAs in PAH, the lncRNA-mRNA Co-Expression network was constructed on the basis of dysregulated lncRNAs and mRNAs. After a strict screening process (PCC > 0.99), 30 differentially expressed lncRNAs were selected for the Co-Expression network. According to correlation coefficient, a total of 262 pairs of interaction relationships between lncRNAs and mRNAs were identified (Figure 3). The interactive mRNAs included LGALS3, PDGFC, SERPINA1, NFIL3, and so on, which could act as key regulatory factors in biological processes predicted by bioinformatics analysis above. The results partly uncovered the hidden competing endogenous RNA (ceRNA) mechanisms, and suggested these co-expressed lncRNAs might play important roles in the pathogenesis of PAH.

### Validation of Aberrantly Expressed Long Non-Coding RNAs and mRNAs

**FIGURE 4:**
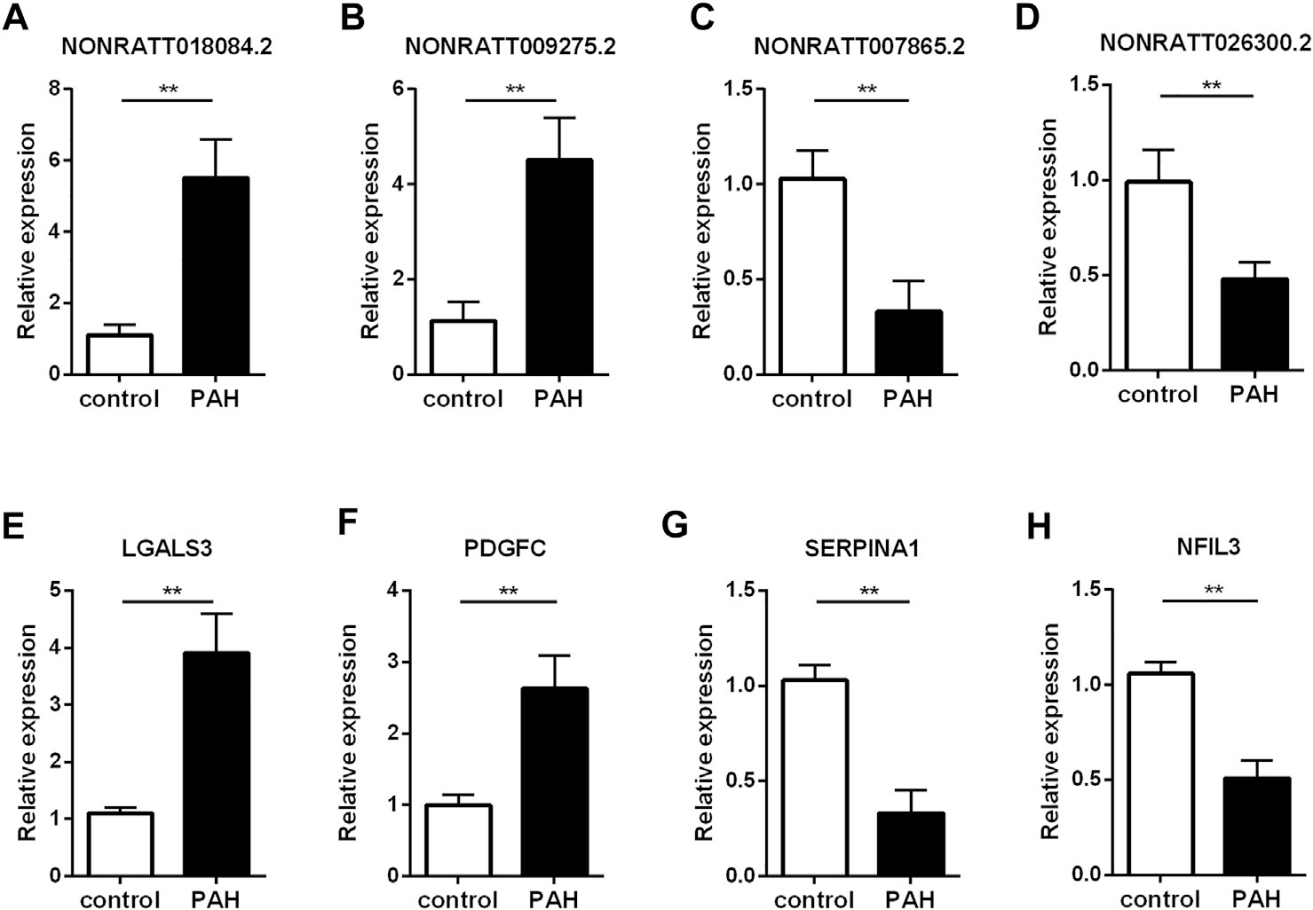
Verification of the critical dysregulated lncRNAs and mRNAs by qRT-PCR. **(A–D)** The expression levels of four lncRNAs (NONRATT018084.2, NONRATT009275.2, NONRATT007865.2 and NONRATT026300.2). **(E–H)** The expression levels of four mRNAs (LGALS3, PDGFC, SERPINA1, and NFIL3). GAPDH was used as the internal control. The data are expressed as the mean ± SD (*n* = 3). **p < 0.01 vs. the control group.

To validate the reliability of the sequencing results and ensure some key regulatory genes, we carried out qRT-PCR assay to verify the expression levels of lncRNAs and mRNAs in another three MCT-PAH rats and control rats. Four mRNAs were picked from the co-expression network because they were involved in the enriched GO terms or pathways, and were considered as key molecules for immune response (Arora et al., 1978; Pohlers et al., 2006; Kashiwada et al., 2010; Schroeder et al., 2020). And the verified lncRNAs are co-expressed with the known genes involved in the enriched GO terms or pathways. Among the four lncRNAs, NONRATT018084.2 and NONRATT009275.2 were found to be significantly up-regulated, whereas NONRATT007865.2 and NONRATT026300.2 were down-regulated in MCT-PAH rats (Figures 4A–D, *p* < 0.01). Then, four key differentially expressed mRNAs, involved in the biological processes identified by bioinformatics results above, were subjected to qRT-PCR. LGALS3 and PDGFC were significantly up-regulated, whereas SERPINA1 and NFIL3 were significantly down-regulated (Figures 4E–H, *p* < 0.01). These data were consistent with the high-throughput sequencing data. Hence, the reliability of sequencing results were confirmed, and several key functional genes were further identified.

### Regulation of Macrophage Polarization by Long Non-Coding RNA NONRATT009275.2

**FIGURE 5:**
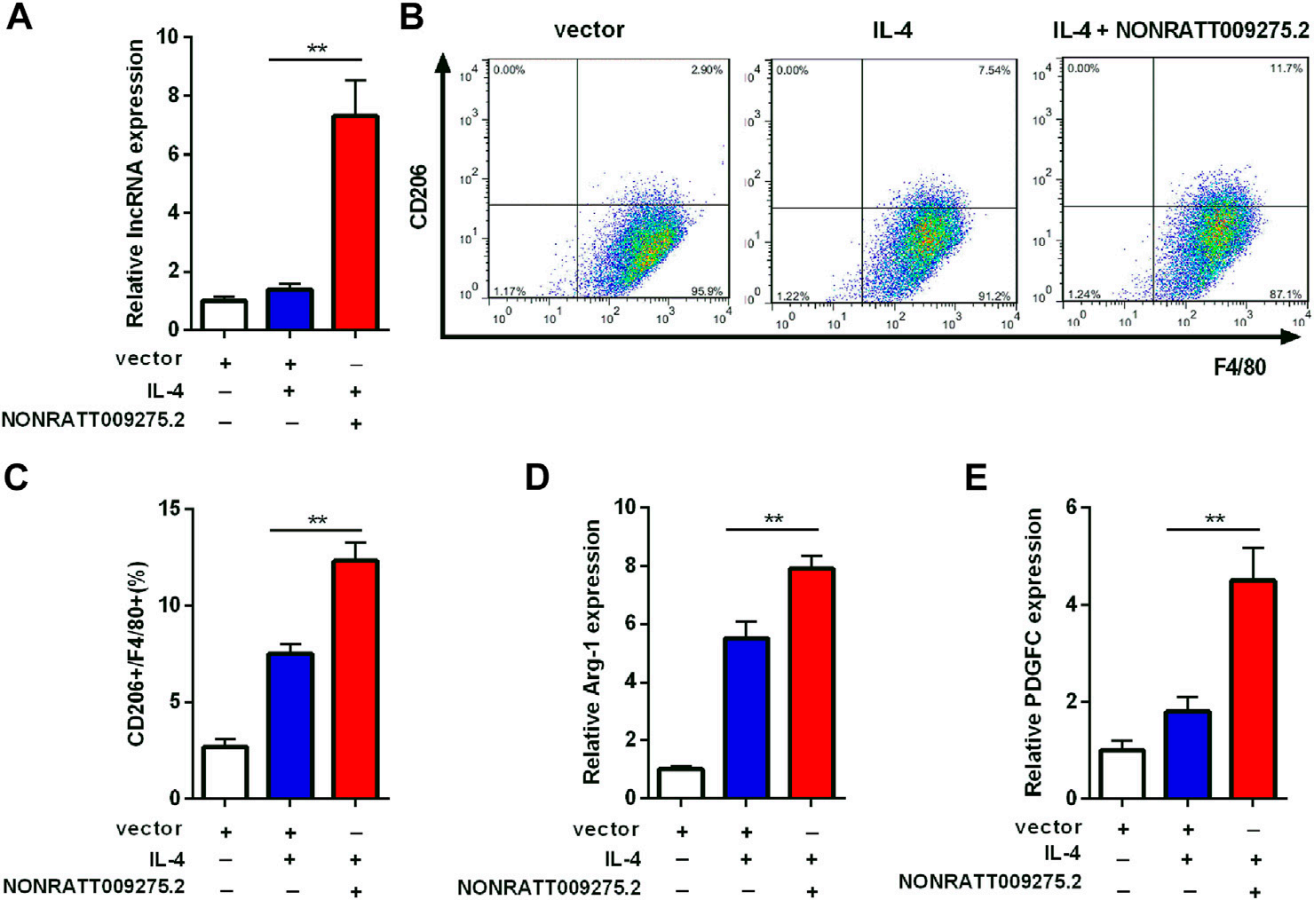
LncRNA NONRATT009275.2 facilitated macrophage polarization to M2 type. **(A)** The expression level of NONRATT009275.2 in transfected BMDMs. **(B)** The macrophage polarization was detected by flow cytometry. **(C)** The F4/80 and CD206 positive cells were quantified. **(D,E)** The Arg-1 and PDGFC mRNA expression were tested by qRT-PCR. GAPDH was used as the internal control. The data are expressed as the mean ± SD (*n* = 3). **p < 0.01 vs. the IL-4 + vector group.

To further confirm that the newly discovered targets are involved in immune inflammatory response, we conducted a preliminary cell function experiment. After transfection of LncRNA NONRATT009275.2 plasmids, NONRATT009275.2 was significantly up-regulated in BMDMs and transfection efficiency was verified (Figure 5A). Then macrophage polarization was detected by flow cytometry and qRT-PCR. Compared with the control group (vector + IL-4), NONRATT009275.2 enhanced macrophage polarization to M2 type in IL-4-stimulated BMDMs (Figures 5B,C). The Arg-1 mRNA expression, as a M2 marker, was also up-regulated (Figure 5D). According to the above lncRNA-mRNA co-expression network, we tested the expression of PDGFC with qRT-PCR, and found the PDGFC mRNA was up-regulated with the overexpression of NONRATT009275.2 (Figure 5E)*.* Taken together, these data indicated that lncRNA NONRATT009275.2 could facilitate macrophage polarization to M2 type.

## Discussion

Understanding the role played by altered ncRNAs in the development of PAH is an area of intense interest. There are more and more evidences suggesting that lncRNAs are pivotal regulators of various pathophysiological processes, including, but not limited to, cell function regulation (Leeper and Maegdefessel, 2018), immune and inflammatory responses (Haemmig et al., 2018), and vascular angiogenesis (Yu and Wang, 2018). With the development of new research techniques, an increasing number of microarrays and high-throughput sequencing platforms have been finished for clarifying differential expression profiling in PAH.

As far as we know, this study is the first to identify the expression profiles of lncRNAs in lung tissues from MCT-PAH rats. It has been reported that the profile of lncRNAs was significantly changed in the lungs of hypoxic pulmonary hypertension rats (Wang et al., 2016). A recent microarray analysis, in pulmonary arteries (PAs) of PAH rats induced by MCT, indicated that a total of 24 lncRNAs and 82 mRNAs were aberrantly expressed (Sun et al., 2019). In PAH rats with right ventricle (RV) failure induced by acute inflammation, 169 lncRNAs and 898 mRNAs were found to be aberrantly expressed in RV myocardium (Cao et al., 2018). In addition, there were some studies involved in human specimens. For chronic thromboembolic pulmonary hypertension patients, differential expression of 185 lncRNAs was found out in PAs (Gu et al., 2015). However, in plasma of 8 PAH patients, 84 candidate lncRNAs were either not expressed or little expressed with no significant difference (Schlosser et al., 2016). In fact, the expression profiles of lncRNAs in PAH are quite different among the above researches, which focused on some kind of tissue, or a certain pathogenic factor. Since PAH is a complicated pathophysiologic process, it is necessary to demonstrate the changes of different tissues and the effects of varied pathogenic factors. Our study mostly focused on the target organ and microenvironment, so lungs were chosen as the detected tissues for PAH. In our work, the profile of lncRNAs in lungs from MCT-PAH rats was firstly identified by high-throughput sequencing, which was different from the profiles of other reports. On the whole, 295 up-regulated and 264 down-regulated lncRNAs were confirmed to have aberrant expression, which contained a large number of novel lncRNAs.

LncRNAs have essential roles in the occurrence and development of PAH. Apoptosis and excessive proliferation and migration are dominant mechanisms of vascular remodeling induced by pulmonary artery smooth muscle cell (PASMC) dysregulation, a classical determinant in the pathogenesis of PAH (Tajsic and Morrell, 2011). It was reported that lncRNA H19 upregulated Angiotensin II receptor Type 1 by sponging let-7b, subsequently facilitating the development of PAH by up-regulating PASMC proliferation. And the lncRNA H19 knockout prevented pulmonary artery remodeling in MCT-PAH animal models (Su et al., 2018). Sun et al. (Sun et al., 2019) reported that overexpression of NONRATT015587.2 accelerated PASMC proliferation, upregulated Hif-1α expression, whereas its silencing promoted PASMC apoptosis. Similar to PASMC dysregulation, pulmonary artery endothelial cell (PAEC) dysregulation and its mesenchymal transition (EndMT) can also result in vascular remodeling related to PAH pathogenesis (Cho et al., 2018). A new lncRNA n342419, also named MANTIS, was down-regulated in lungs of PAH patients, which low expression compromised the reparative ability of PAECs, thereby perpetuating vascular remodeling (Leisegang et al., 2017). In our study, several previously reported lncRNAs were also found. More importantly, the profiles of lncRNAs and mRNAs from lungs in this study were able to provide more comprehensive candidate genes for improving the regulatory network of PAH.

The functional analysis demonstrated the potential roles of lncRNAs, which mainly enriched in inflammatory and immune response. Several key genes, including LGALS3 (Schroeder et al., 2020), PDGFC (Liu et al., 2019), NFIL3 (Kashiwada et al., 2010) and so on, were further verified in this study. Activation of immune cells and release of proinflammatory factors in system and microenvironment could be powerful contributors to numerous cardiovascular diseases. In recent years, greater attention has focused on the perivascular inflammation in patients with all forms of PAH (Rabinovitch et al., 2014; Gu et al., 2015). In fact, the inflammatory process is bound up with metabolic changes of blood vessels and inflammatory cells. Some lncRNAs have been proven to be involved in vascular inflammatory response. LncRNA MALAT1 up-regulated glucose-induced inflammatory mediators IL-6 and TNF-α by activating serum amyloid antigen 3 in HUVECs (Puthanveetil et al., 2015). Additionally, lncRNA MALAT1 could rescue TGF-β Receptor Type II from post-transcriptional suppression via sponging off miR-145 in endothelial progenitor cells (Li et al., 2019). NKILA, a cytoplasmic lncRNA, was reported to have an interaction with NF-κB/IκB complex, indicating a role of NKILA as a crucial moderator to protect the endothelium from inflammatory lesions and associated vascular disorders (Zhu et al., 2019). Recently, proinflammatory cytokine-induced lncRNA Giver was newly identified, which upregulated IL-6, CCL-2, and TNF to augment oxidative stress level and cell proliferation in vascular smooth muscle cells (Das et al., 2018). In this study, we found the newly discovered PAH related lncRNA, NONRATT009275.2, facilitated macrophage polarization to M2 type. To some extent, our preliminary experiment confirmed the dysregulated expression profiles are involved in inflammatory immune response.

In addition to inflammatory response, our functional analysis still found out that renin-angiotensin system and circadian rhythm participated in pathological process of PAH. Renin-angiotensin activity, as an important pathogenesis of hypertension, was also reported to be increased in PAH patients, and its inhibition by losartan was beneficial in experimental PAH (de Man et al., 2012). As far as we know, there are no studies involved in the relationship between circadian rhythm and PAH. Hence, our bioinformatics analysis could provide a new research direction for PAH. As important regulators of vascular pathophysiology, the role of lncRNAs in PAH remains unclear and further research is needed.

The original purpose of this sequencing was to have a holistic view of gene expression about PAH, and find more possible pathogenesis, not only inflammation or immunity. However, after bioinformatics analysis, we were surprised to find out that the dysregulated genes were mainly enriched in immunity, which indicated that immunity could play a major role in the pathological process of PAH. Then, we focused on immunity and inflammation and did a preliminary verification experiment. In fact, the lung tissue is a broad concept, including multiple cell types. While the lung tissue sequencing brought more potential pathological mechanisms, it also had some limitation, including the poor cell specificity. The levels of inflammatory factors and the effects on PAH *in vitro* and *in vivo* should be explored in further research.

In summary, this study, for the first time, analyzed the lncRNA and mRNA high-throughput sequencings in lung tissues of MCT-PAH rat models. Through a series of bioinformatics analyses, numerous aberrantly expressed lncRNAs and mRNAs were identified, and their potential roles in the pathogenesis of PAH were further predicted. We also constructed the co-expression network to expand the understanding of gene interactions. Several key regulatory genes were confirmed using qRT-PCR. In the end, we preliminarily verified the pathophysiological mechanism of lncRNA NONRATT009275.2 involved in PAH. Our research provided more comprehensive candidate genes and potential roles in modulating the pathogenesis of PAH, which might serve as valuable biomarkers and therapeutic targets, and laid the initial foundation for further mechanism study in PAH.

## Data Availability

The datasets presented in our study can be found in online repositories. The names of the repository/repositories and accession number(s) can be found below: Sequence Read Archive database (https://www.ncbi.nlm.nih.gov/bioproject/PRJNA732522/).
