# Supplementary material for: Profiling and Molecular Mechanism Analysis of Long Non-Coding RNAs and mRNAs in Pulmonary Arterial Hypertension Rat Models
Source: Front Pharmacol. 2021 Jun 29;12:709816. doi: 10.3389/fphar.2021.709816 (PMC8277419; doi:10.3389/fphar.2021.709816)
Supplement: Supplementary file 1 [file DataSheet1.docx]

***Supplementary Material***

**

**

**FIGURE S1.** Characteristics of dysregulated lncRNAs in PAH. **(A)** The types of dysregulated lncRNAs, which were mainly divided into intergenic lncRNAs (u)，intronic lncRNAs (i), anti-sense lncRNAs (x) and sense-overlapping lncRNAs (o). **(B)** The number of exons in dysregulated lncRNAs. **(C)** The length of dysregulated lncRNAs.


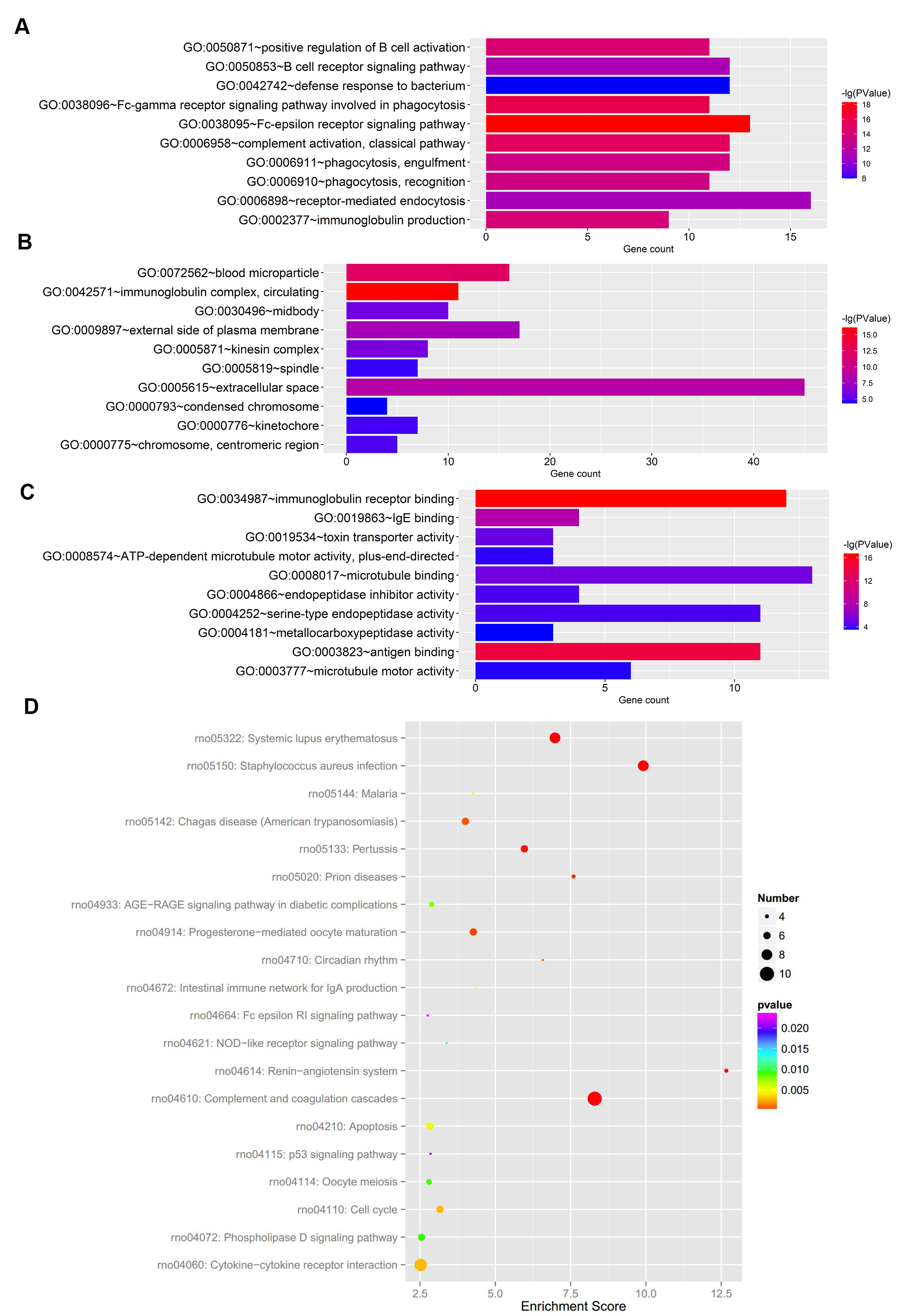


**FIGURE S2.** GO enrichment and pathway analysis for up-regulated mRNAs. Top 10 enriched GO terms of up-regulated mRNAs were presented according to **(A)** biological process, **(B)** cellular component and **(C)** molecular function, respectively. **(D)** KEGG pathway analysis of up-regulated mRNAs in PAH.


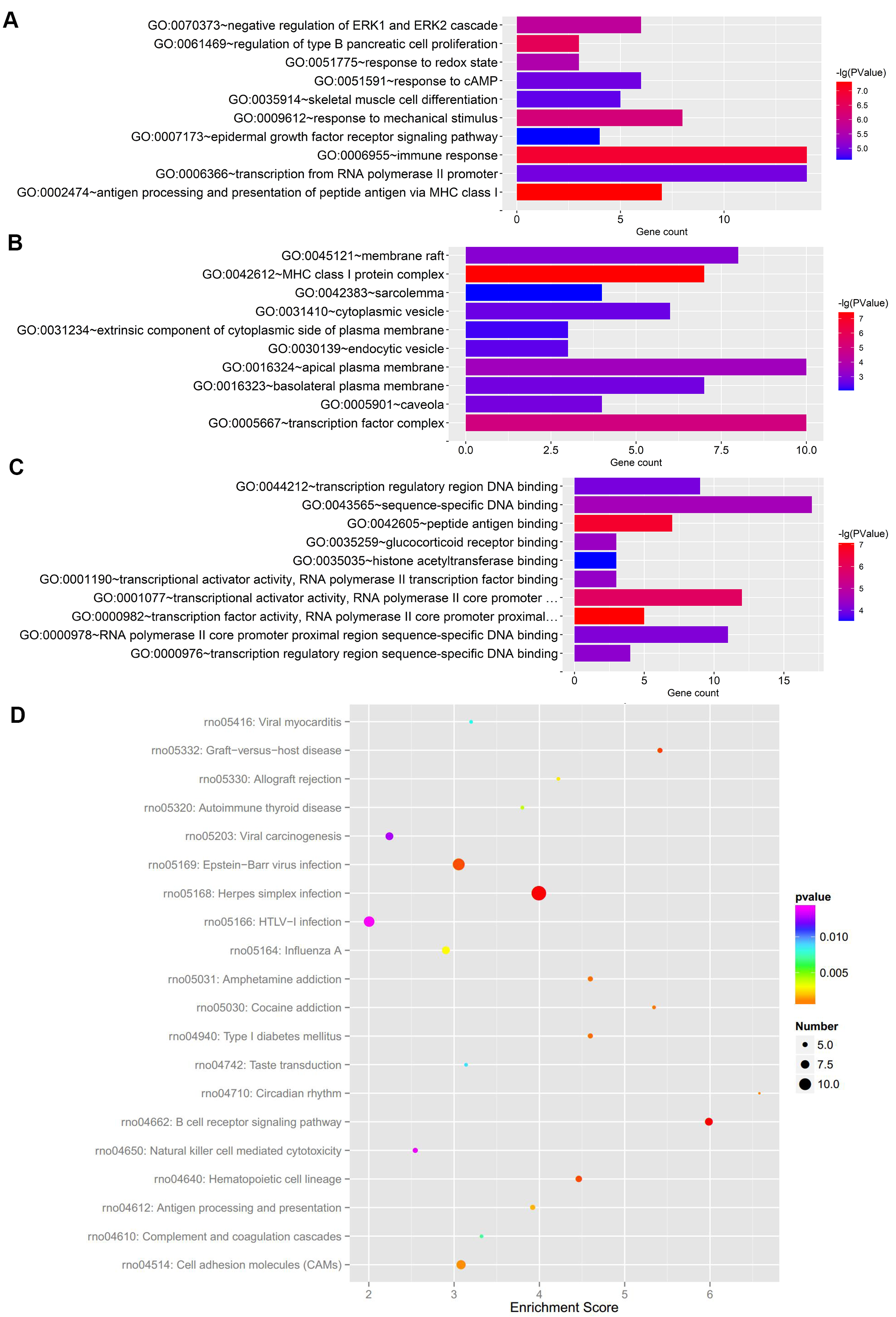


**FIGURE S3.** GO enrichment and pathway analysis for down-regulated mRNAs. Top 10 enriched GO terms of down-regulated mRNAs were presented according to **(A)** biological process, **(B)** cellular component and **(C)** molecular function, respectively. **(D)** KEGG pathway analysis of down-regulated mRNAs in PAH.

**Table S1.** Primers for qRT-PCR

| ID | Primer |
| --- | --- |
| NONRATT018084.2 | forward: 5'-CTGCATCTCTGGGAGACAAA-3' |
|  | reverse: 5'-CACGTTGCTGATGCTGAATG-3' |
| NONRATT009275.2 | forward: 5'-GGGTTGTGGTTTGGAACTATCT-3' |
|  | reverse: 5'-CCAAAGAATCATTCACACAGGTATATG-3' |
| NONRATT007865.2 | forward: 5'-GTCCTTTGTTTCTGCTCCTCTA-3' |
|  | reverse: 5'-CTACTTGGCTCCAGACTGTTC-3' |
| NONRATT026300.2 | forward: 5'-GTGGATTAGAGCCACCATGAA-3' |
|  | reverse: 5'-CCACAGCTCCTCACCAAATAG-3' |
| LGALS3 | forward: 5'-GGCCACTGATTGTGCCTTAT-3' |
|  | reverse: 5'-GAAGCGTGGGTTAAAGTGGA-3' |
| PDGFC | forward: 5'-CAGCAAGTTGCAGCTCTCCA-3' |
|  | reverse: 5'-GACAACTCTCTCATGCCGGG-3' |
| SERPINA1 | forward: 5'-AATGGGGCTGACCTCTCC-3' |
|  | reverse: 5'-GTCAGCACAGCCTTATGCAC-3' |
| NFIL3 | forward: 5'-AAGCTTTGGACAGTGAGTTCG-3' |
|  | reverse: 5'-TTACCTGGAGTCCGAAGCCG-3' |
| Arg-1 | forward: 5'-CAGAAGAATGGAAGAGTCAG-3' |
|  | reverse: 5'-CAGATATGCAGGGAGTCACC-3' |
| GAPDH | forward: 5'-TCGCCATCTTCAAGTCGGG-3' |
|  | reverse: 5'-AGGGGCACCATCTTTTGCAG-3' |

qRT-PCR: quantitative real-time PCR.
